# Scottish Metropolitan Veterinary Medical Society

**Published:** 1887-01

**Authors:** 


### SCOTTISH METROPOLITAN VETERINARY MEDICAL SOCIETY.

The quarterly meeting of this Society was held in the London Hotel, Edinburgh, on Nov. 22. There was a good attendance of members. Professor Williams occupied the chair.

Mr. Rutherford, Edinburgh, introduced a discussion on the suppression of contagious and infectious diseases in the lower animals.

Referring to pleuro-pneumonia, Mr. Rutherford said they had heard a great deal about this disease of late. It was much more common in Scotland and England just now than it had been for seven or eight years. Some authorities proposed killing. He protested against any such proposal in the matter. The disease was as easily handled as any disease he was aware of. Slaughtering, undoubtedly, put an end to the disease, but by this method they involved stockowners in an enormous expense, and it does not necessarily kill out the contagion. And it sometimes happened that they had to kill cattle which it was impossible to replace, while they at the same time were in a manner destroying public money. He held, therefore, that slaughtering was impolitic, and, in view of what they now knew of inoculation, was entirely unnecessary. His proposition was to make inoculation a compulsory operation along with slaughtering, where the disease was known to exist. By that combined method the disease would soon disappear.

Professor Macfadyean said that he would endeavor to controvert several of the views just expressed by Mr. Rutherford; and although he might do so in firm language, he would do it with the greatest good nature. He would confine his remarks chiefly to the pleuro-pneumonia question. Professor Macfadyean said they might recognize four methods of dealing with the disease. The first of these was the method of general compulsory inoculation of all the bovine animals in this country. No sane man would recommend that method, for the very good reason that the aggregate deaths from the operation would far exceed the present annual loss from pleuro-pneumonia. At the same time, he said, this method, if it conferred an immunity from pleuro-pneumonia equal to that conferred by Pasteur's vaccine against anthrax, might reasonably be expected to ultimately suppress pleuro-pneumonia. The second method of dealing with the disease was that advocated by Mr. Rutherford, viz., slaughter of the affected animals and compulsory inoculation of all animals that had been exposed to infection. The advocates of this method claimed for it that it would, if put into general operation, ultimately eradicate the disease; for, say they, all the animals already affected would be slaughtered, the healthy would immediately be protected by inoculation, and the virus that already contaminated the building, being unable to find the system of an animal in which to propagate itself, would soon die a natural death. He would endeavor to state clearly his objections to that train of reasoning, or of assumptions, as he would prefer to call it. Would all the animals already affected be slaughtered when the veterinary surgeon was called in to treat an outbreak? He said no, to that question. He believed that the advocates of inoculation said that they had two crucial tests by which they could weed out the animals already attacked. The first of these was thermometric observation; the second was inoculation itself. With regard to the first of these, he was going to put a question to Mr. Rutherford, or any other member of his way of thinking, viz.: What were the distinctive characters of the temperature chart of pleuro-pneumonia? Unless he was told explicitly in what respects it differed from other conditions of abnormal temperature —simple pleurisy or pneumonia, tubercle, or acute indigestion, for example—he would maintain that some of them had been guilty of making a loose statement that might find credence with the lay public, but would not be accepted by that meeting of professional men. He denied, moreover, the accuracy of the statement that the affected animals would be indicated to them by watching the effects of inoculation, since those already affected would not 'take' the inoculation. His own observation enabled him to say that a cow already affected with pleuro-pneumonia might 'take' the inoculation, and show precisely the same inoculative lesions in the tail as an otherwise healthy animal. To prove his contentions and show that those were not mere fanciful objections, Professor Macfadyean cited cases in which he had made post-mortem examination of cows inoculated one month previously by Mr. Rutherford. These cows showed the usual inoculative lesions in the tail, and at the same time they had pleuro-pneumonia in their lungs. Again, he held that any statement to the effect that the virus in a contaminated building would die a natural death, was a mere assumption; for on that point they could have no accurate information until the micro-parasite of the disease had been isolated and cultivated artificially. Another question that he would like to notice here was this: Does inoculation of the tail produce pleuro-pneumonia? He held that careful reasoning compelled them to answer in the affirmative. He had now stated what appeared to him to be serious objections to the practice of inoculation, and he invited the advocates of the practice to explain them away if they could. He was quite open to conviction, but those who favored inoculation must not be angry if he demanded a reason for the faith that was in them. The third method of dealing with pleuro-pneumonia was that of compulsory slaughter of the affected, and isolation of those that had been exposed to infection. That method now stood discredited by its results. The fourth method was that of compulsory slaughter of the entire herd—both the affected and those that had been exposed to infection. He unhesitatingly expressed his belief that this plan, if put into operation, would in a short time, and at a trifiing cost, as compared with the accumulated annual loss from pleuro-pneumonia, stamp out this disease just as effectually as it had stamped out rinderpest and sheep-pox.

He repeated what he had frequently before stated, that in his opinion it was not a question of suppression but of prevention, and that they would never eradicate pleuro-pneumonia from the country by inoculation, other and more radical measures must be put in force; and as to the failure of the Netherlands Government to get rid of it by inoculation, being attributable to faults in the method of operating, he could only say that if the father of inoculation in European countries, viz.: Willems, of Hasselt, failed in suppressing the disease, with all the power of the Government at his back and with the full control and supervision of the practice, no individual or body of individuals in this or any other country were likely to be successful in that direction. If they were sincerely desirous of ridding the country of this scourge they should not advise the adoption of half measures, and if there is at the outset some sacrifice in the adoption of radical measures, the loss would be a hundred times repaid by the freedom which would be restored to trade in cattle, both at home and for exportation.

Professor Williams, in winding up the discussion, thought inoculation had done a great deal of good. He was not prepared, however, to give a decided opinion till some large experiment had been made at the public expense.

