# Correction

**Published:** 1887-04

**Authors:** 


Correction.—In report of Scottish Metropolitan Association meeting, in January No. of Journal—the remarks made on page 95 after line 11 were made by Professor Walley not Professor McF.

W. H. Harbaugh, V. S., of Richmond, Va., graduate of Ontario, desires us to state that he is not the Dr. Harbaugh who met his death in Knoxville, Tenn.

